# Novel arthrocentesis approaches to the carpal joint of the Dromedary Camel (*Camelus dromedarius*)

**DOI:** 10.1038/s41598-022-16801-3

**Published:** 2022-07-27

**Authors:** Fransina Christina King, Adnan Aldarwich, Maha Hammoud, Robert Barigye, Turke Shawaf, Ahmad Al Aiyan

**Affiliations:** 1 Department of Veterinary Medicine, College of Food and Agriculture, United Arab Emirates University, Al Ain, UAE; 2 Department of Clinical Sciences, College of Veterinary Medicine, King Faisal University, Al-Hasa, Saudi Arabia

**Keywords:** Anatomy, Musculoskeletal system

## Abstract

The knowledge gap regarding the topography and anatomy of the dromedary’s carpal joint must be bridged to improve diagnostic and treatment procedures such as ultrasonography, arthrocentesis, and arthroscopy. Thirty-five distal forelimbs were harvested from 21 dromedaries and studied through gross dissection, casting, ultrasonography, and computerized tomography. Representative three-dimensional models of the joint cavities, recesses, and pouches were obtained using various casting agents. The safety and feasibility of different arthrocentesis approaches were evaluated. This study provides a detailed description of dorsally located joint recesses and palmarly located joint pouches. The dorsomedial and dorsolateral approach is recommended for arthroscopy and arthrocentesis of the radiocarpal and intercarpal joint when the carpus is flexed. However, caution must be exercised during these approaches to prevent needle injury to the articulating cartilage. Caution is necessary to prevent the formation of inadvertent communication between the dorsally located tendon sheaths and joint cavities. Arthrocentesis via the lateral approach to the lateropalmar pouch is the most favourable approach for the radiocarpal joint. A subtendinous synovial bursa was found between the lateropalmar pouch of the radiocarpal joint and the extensor carpi ulnaris muscle. The subtendinous synovial bursa must be considered during the lateral arthrocentesis approach. The palmar approach is not recommended for arthrocentesis due to the high risk of injury to nerves, veins, and arteries located palmarly.

## Introduction

Owing to their incredible ability to adapt to harsh climate conditions, dromedaries play a considerable role in livestock production in the Middle East^1,2^. They are also excellent racing animals, which has rendered them invaluable in the Arab social culture^2–4^. Despite their rugged image, camels suffer from various illnesses, particularly lameness, the fourth primary affliction associated with arthritis^1,3^. The carpus region is highly susceptible to bursitis, a secondary inflammatory response to repeated trauma or brucellosis^5^. This joint is especially crucial in camels due to their unique biomechanical action when they sit down, stand up, or remain in a recumbent position^6^.

Lameness in camels causes substantial economic losses to farmers and owners due to decreased production, impaired reproductive performance, and decreased physical performance. The limbs of camels exhibit unique anatomical developments^4,7–10^. This complicates the comparison of lameness patterns observed in camels with those commonly noted in bovine and equine species^3^. In camels, fundamental knowledge regarding the anatomy and topography of healthy unaffected joints is essential for the identification of pathological abnormalities^6,11^. A definitive diagnosis is reached for most articular-related problems by performing a physical lameness examination. This evaluation is supported by radiographic, ultrasonographic, or computerized tomography (CT) imaging to identify the anatomical origin of the lameness^12^. Arthrocentesis is another valuable technique for diagnosing and treating affected joints^4^. However, incorrect needle placement during arthrocentesis can cause severe complications (e.g. acute aseptic arthritis). Such complications can develop shortly after an intra-articular injection due to articular cartilage damage and are exceptionally difficult to treat, potentially leading to joint degeneration^13^.

Currently, the scientific literature on arthrocentesis in camels is limited^4^, and far less published research is available on the carpal joint of camels compared to equines and bovines^2,14,15^. The knowledge gap regarding the detailed anatomy of joint structures can pose challenges to the diagnosis of abnormalities. A comprehensive understanding of the anatomy of these joints is required to enhance current arthrocentesis techniques and create optimal arthrocentesis approaches to the dromedary’s carpal joint.

The present study aimed to describe the basic anatomy and topography of the carpus, including the joint cavities, joint capsule, and synovial pouches of the non-pathological carpal joint of the dromedary. The different methods utilized in this investigation included casting, gross dissection, ultrasonography, and computed tomography imaging. This study also aimed to provide guidelines for performing ultrasonography of the carpal joint. Furthermore, current arthrocentesis techniques were re-evaluated in this study to develop and provide guidelines for the safe and feasible arthrocentesis of the dromedary carpal joint.

## Methods and materials

This study adhered to the Research Ethics Policy and was approved by the Animal Research Ethics Committee of the United Arab Emirates University (approval number: ERA_2020_6088). The reported experiments complied with the Animal Research: Reporting of In vivo Experiments Guidelines of the United Arab Emirates University.

### Sample collection

The distal forelimbs used in this study were collected from two local abattoirs located in Abu Dhabi, UAE (Al Khazna and Al Bawadi). Post slaughter, 35 distal forelimbs were harvested from 21 dromedaries. The distal forelimbs were amputated 15 cm above the carpal joint and harvested from male and female animals aged 1–4 years. All animals were free from any pre-existing lameness or joint illness.

### Arthrocentesis

Thirteen radiocarpal and intercarpal joints were injected from a dorsomedial approach, and another 13 radiocarpal and intercarpal joints were injected from a dorsolateral approach. Moreover, 26 carpometacarpal joints were indirectly injected via their connection with the intercarpal joint. Three radiocarpal joints were injected from a lateral approach, and another three were injected from a palmar approach. Six carpometacarpal joints were injected from a palmar approach, which led to an indirect injection of the intercarpal joints via the joint connection.

During the dorsomedial approach, the radiocarpal joint was flexed. The extensor carpi radialis tendon, distal surface of the radius, and proximal surface of the proximal row of carpal bones were identified through palpation. An evident depression was formed in this area. A 14-gauge needle attached to a closed three-way-stop-cock catheter was inserted 5 mm medial to the extensor carpi radialis tendon (Fig. 7).

For the dorsomedial approach of the intercarpal joints, the joint was flexed, and the extensor carpi radialis tendon, distal surface of the proximal row of carpal bones, and the proximal surface of the distal row of carpal bones were palpated. A clear depression was formed in this area, and the needle (attached to a three-way-stop-cock catheter) was inserted into the depression, 5 mm medial to the extensor carpi radialis (Fig. 7).

The carpus was flexed during the dorsolateral approach of the radiocarpal joint and intercarpal joint. The injection site for the radiocarpal joint was identified by palpating the depression formed lateral to the extensor carpi radialis, distal to the radius and ulna, and proximal to the proximal row of carpal bones. The needle was inserted 5–6 mm lateral to the extensor carpi radialis tendon (Fig. 8). The injection site of the intercarpal joint was identified by palpating the depression formed between the extensor carpi radialis, distal surface of the proximal row of carpal bones, and the proximal surface of the distal row of carpal bones. The needle attached to a three-way-stop-cock catheter was inserted 5–6 mm lateral to the extensor carpi radialis tendon (Fig. 8).

### Casting

Twenty radiocarpal joints and 20 intercarpal joints were injected with EasyFlo 60 Liquid Plastic (Polytek Development Corp, Easton, Pennsylvania, USA). Six radiocarpal joints and six intercarpal joints were injected with Globalsil AL 20 Flexible Mould Rubber (GLOBALCHIMICA S.r.l., Piedmont, Turin, Italy). Five radiocarpal joints and five intercarpal joints were injected with Eastcoast Epoxy Resin (East Coast Resin, Brooklyn, New York, USA). Three radiocarpal joint and three intercarpal joints were injected with Gulf Guard Epoxy, Abu Dhabi, United Arab Emirates. The casting agents were prepared according to the instructions provided by the manufacturer. Blue dye was used to colour the casting agent injected into the radiocarpal joint, and red dye was used to colour the casting agents injected into the intercarpal joint.

The synovial fluid was extracted using the above-described injection approaches from the various joint cavities. The casting materials were injected gradually into the joint cavity with manual pressure, using syringes (20–60 ml), until resistance was felt. The needle and catheter were not removed to prevent the backflow of the casting material. Post casting, the samples were kept at room temperature for 4 h, followed by storage for 24 h at 2–5 °C. Samples were stored for ≥ 24 h before dissection or 48 h before commencing maceration. The skin, muscles and tendons were removed from the non-dissected sample in preparation for the maceration procedure. Samples were subsequently placed in a 2% sodium bicarbonate and water solution at 40 °C for 48 h. The samples were removed, and the sodium bicarbonate solution was discarded. The saturated soft tissue was manually removed, and the samples were placed into a freshly mixed 2% sodium bicarbonate and water solution at 40 °C for 72 h. The specimens were removed and allowed to dry for 24 h. Gluing and refixing of any structures of the specimens were performed by a lab specialist.

### Dissection

Seven distal forelimbs were dissected to examine the impact of the needle on surrounding joint structures during arthrocentesis. The potential joint cavity communications with surrounding tendon sheaths, joint capsules, and the joint recesses and pouches were extensively examined during the dissection. The samples were macerated after dissection, and data were collected as discussed above. Five distal forelimbs were dissected to study the gross anatomy of the space between the articulating bone surfaces, range of joint flexion, and exposure of the joint cartilage surface during flexion.

### Ultrasonography

Ultrasonography was conducted using eight specimens. A MyLab^TM^OmegaVet (series 7400 model) (Esaote, Florence, Italy) ultrasound device, combined with an Esaote L4-15 (4–15 MHz; 47 mm) linear transducer, was used. The equine tendon superficial and deep presenting were selected alternatively to capture the best quality images. The carpus region was shaved, the limbs were placed horizontally on the table, and a generous amount of ultrasound gel was used.

The dorsomedial aspect of the carpal joint was investigated in an extended and flexed position, and the probe was placed longitudinally, medial to the extensor carpi radialis. The dorsal aspect of the carpal joint was investigated in an extended and partially flexed position, and the probe was placed in a longitudinal orientation, directly on top of the extensor carpi radialis tendon. The dorsolateral aspect of the carpus was investigated by placing the linear probe lateral to the extensor carpi radialis tendon.

The carpal joints’ mediopalmar and lateropalmar aspects were investigated by placing the probe medial and lateral to the carpal canal, respectively. The carpal joint was extended, and the probe was placed in a longitudinal orientation for both aspects. The lateral aspect of the carpal joint was investigated in an extended position. For the scans mentioned above, the probe was placed at the level of the epiphysial line of the radius. The probe was moved distally in a parallel line over the carpal joint up to the epiphysial line of the metacarpal bone. For illustrative purposes, the ultrasound images (Figs. 10, 11, 12, 13, 14, 15) were composed of three to four individual ultrasound images using Adobe Photoshop 2022 (Adobe, San Jose, California, USA).

### Computed tomography

The radiocarpal, intercarpal, and carpometacarpal joints were cast using Globalsil AL 20 Flexible Molds Rubber (GLOBALCHIMICA S.r.l., Piedmont, Turin, Italy) mixed with a contrast medium, Omnipaque (350 mg/ml) (GE Healthcare, Chicago, Illinois, USA). The CT images were captured at the Dubai Equine Hospital using an EQUIMAGINE™ multi-modality robotic scanner (Universal Medical Systems, Inc., Solon, Ohio, USA). CT scanning was conducted at 100 kV and 0.50 mAs. Transverse and sagittal series were obtained at a thickness of 0.5 mm.

## Results

### Anatomy and casting

**Figure 1:**
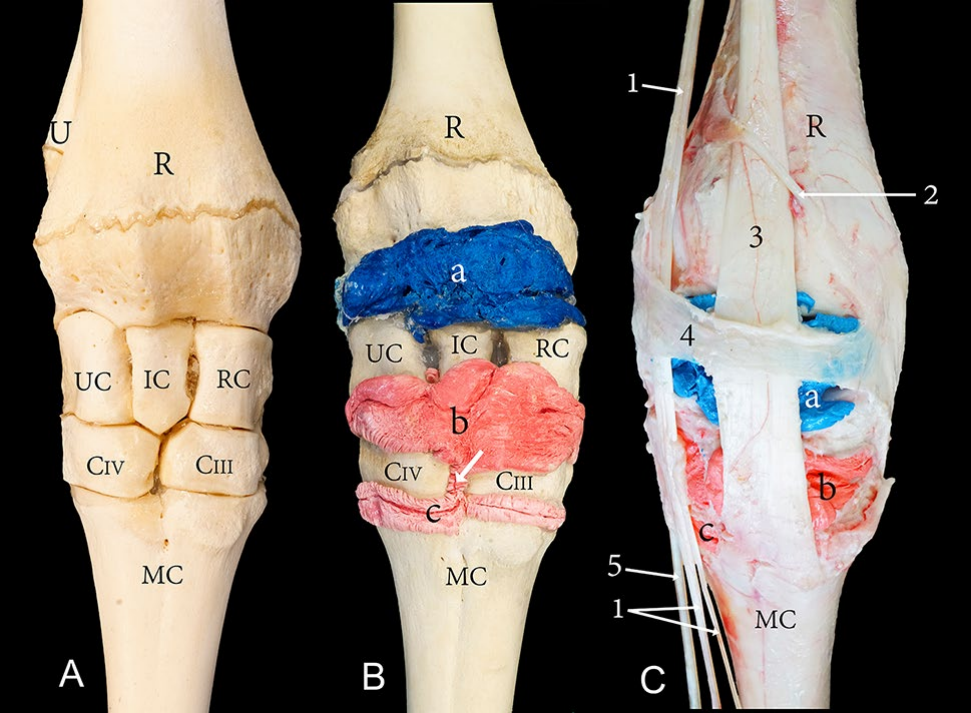
Dorsal view of the right carpus showing (**A**) bones, (**B**) casting, and (**C**) dissection. R, the distal end of the radius; U, the distal end of the ulna; RC, radiocarpal bone; IC, intermediate carpal bone; UC, ulna carpal bone; CIII, carpal bone III; CIV, carpal bone IV; MC, metacarpal bone; a, radiocarpal joint cavity; b, intercarpal joint cavity; c, carpometacarpal joint cavity; 1, common digital extensor tendon; 2, abductor pollicis (digit 1) longus; 3, extensor carpi radialis tendon; 4, extensor retinaculum; 5, lateral digital extensor tendon. The white arrow indicates the communication between the intercarpal and carpometacarpal joint cavity.

The carpal joint of the camel consists of the distal extremity of the radius and ulna, carpal bones, and the proximal extremity of the metacarpal bones. The carpal bones are arranged in two rows. The proximal row includes the radial, intermediate, ulnar, and accessory carpal bones, while the distal row consists of the carpal bones II, III, and IV (Figs. 1A, 3A, 4A, and 5A). The carpal joint has three joint compartments. The proximal joint compartment is the radiocarpal joint, the intermediate joint compartment is the intercarpal joint, and the distal joint compartment is the carpometacarpal joint. In all studied samples, the radiocarpal joint cavity did not communicate with the two distal joint cavities, while the intercarpal joint and the carpometacarpal joint communicated in all investigated samples (Fig. 1B).

**Figure 2:**
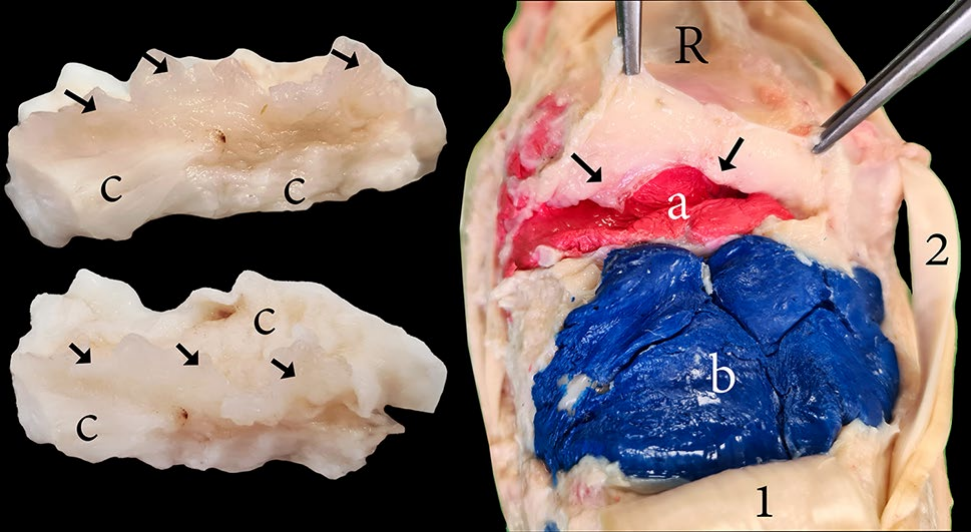
Dorsal view of a left carpal joint showing (a) the casted radiocarpal joint cavity, (b) the casted intercarpal joint cavity, and (c) the internal service of the dorsal joint capsule. R, radius; 1, extensor carpi radialis tendon reflected distally; 2, common digital extensor tendon. The black arrows indicate the folds of the synovial membrane.

Dorsally, the joint capsule of the radiocarpal joint originates from the dorsal surface of the distal part of the radius and extends towards the proximal row of carpal bones where its fibrous layer inserts partly on the dorsal surface of the proximal row of the carpal bones. It continues distally, gives rise to the intercarpal joint capsule, and inserts partly on the distal row of carpal bones. From this point, the fibrous layer continues to give rise to the carpometacarpal joint capsule. It finally inserts on the dorsal surface of the proximal part of the metacarpal bones. Figure 1B indicates the recesses formed by the joint capsule on the radius, the proximal carpal bones, and the distal carpal bones. The internal synovial membrane forms folds, which are exceptionally developed at the level between the articulating bones. These folds were found moulded into the casted material (Fig. 2).

**Figure 3:**
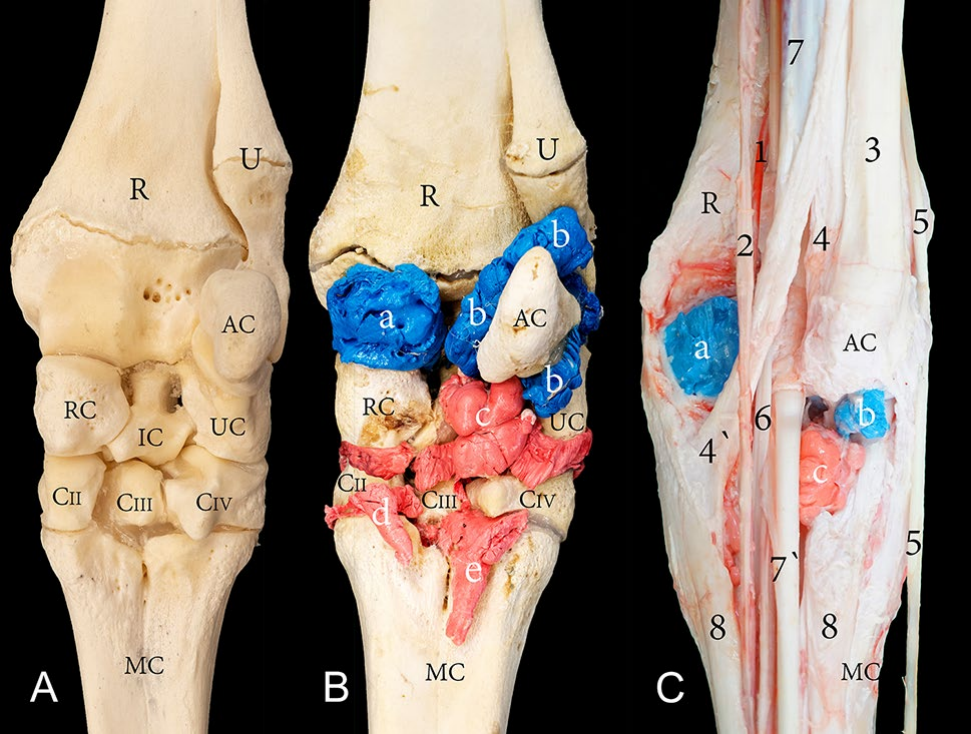
Palmar view of the right carpus showing (**A**) bones, (**B**) casting, and (**C**) dissection. R, the distal end of the radius; U, the distal end of the ulna; AC, accessory carpal bone; RC, radiocarpal bone; IC, intermediate carpal bone; UC, ulna carpal bone; CII, carpal bone II; CIII, carpal bone III; CIV, carpal bone IV; MC, metacarpal bone; a, mediopalmar pouch of the radiocarpal joint; b, lateropalmar pouch of the radiocarpal joint; c, palmar pouch of the intercarpal joint; d, medial pouch of the carpometacarpal joint; e, lateral pouch of the carpometacarpal joint; 1, median artery; 2, median vein; 3, extensor carpi ulnaris tendon; 4, lateral insertion of the flexor carpi ulnaris; 4`, medial insertion of the flexor carpi ulnaris; 5, lateral digital extensor tendon; 6, flexor carpi radialis tendon; 7, humeral head of the flexor digitorum profundis; 7`, flexor digitorum profundis tendon; 8, interosseous medius muscle.

**Figure 4:**
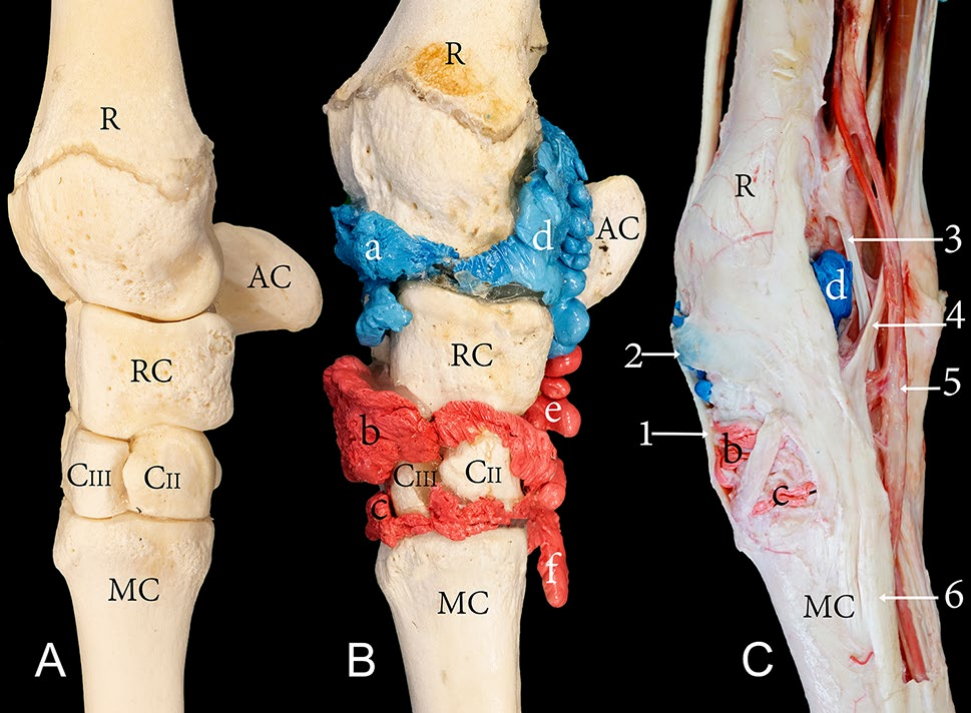
Medial view of the right carpus showing (**A**) bones, (**B**) casting, and (**C**) dissection. R, the distal end of the radius; AC, accessory carpal bone; RC, radiocarpal bone; CII, carpal bone II; CIII, carpal bone III; MC, metacarpal bone; a, radiocarpal joint cavity; b, intercarpal joint cavity; c, carpometacarpal joint cavity; d, mediopalmar pouch of the radiocarpal joint; e, palmar pouch of the intercarpal joint; f, medial pouch of the carpometacarpal joint; 1, extensor carpi radialis tendon; 2, extensor retinaculum; 3, flexor carpi radialis tendon; 4, medial insertion of the flexor carpi ulnaris tendon; 5, median artery; 6, interosseous medius muscle.

**Figure 5:**
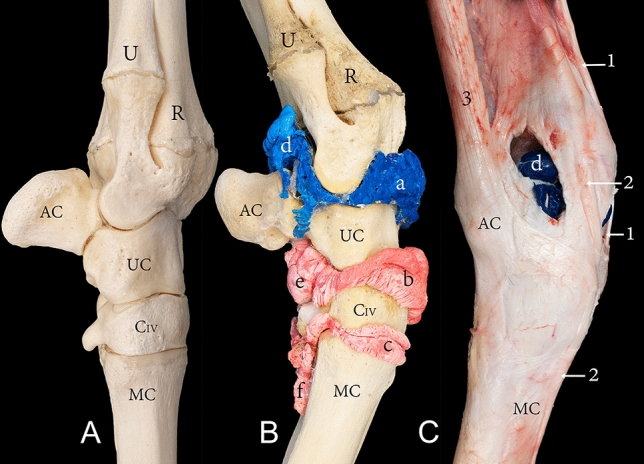
Lateral view of the right carpus showing (**A**) bones, (**B**) casting, and (**C**) dissection. R, the distal end of the radius; U, the distal end of the ulna; AC, accessory carpal bone; UC, ulna carpal bone; CIV, carpal bone IV; MC, metacarpal bone; a, radiocarpal joint cavity; b, intercarpal joint cavity; c, carpometacarpal joint cavity; d, lateropalmar pouch of the radiocarpal joint; e, palmar pouch of the intercarpal joint; f, lateral pouch of the carpometacarpal joint; 1, extensor carpi radialis tendon; 2, lateral digital extensor tendon; 3, extensor carpi ulnaris.

The capsule of the radiocarpal joint forms palmarly two pouches (Fig. 3B,C). The first pouch is located mediopalmar to the radius and the radial carpal bone (Fig. 4B,C). The second pouch is located lateropalmar to the radius and the intermediate carpal bone (Fig. 3B,C). The lateropalmar pouch surrounds the base of the accessory carpal bone. It extends lateropalmarly between the distal end of the ulna and the accessory carpal bone (Figs. 3B, 5B,C). The intercarpal joint forms a palmar pouch at the level of the intermediate carpal bone and carpal bone IV (Fig. 3B,C). At the medial and lateral origin of the interosseous medius muscle, the carpometacarpal joint forms two small pouches which extend distally on the palmar surface of the metacarpal bone (Figs. 3B, 4B, 5B). The small lateral pouch is located at the level of carpal bone IV and the metacarpal bone, and the small medial pouch is located at the level of carpal bone II and the metacarpal bone.

**Figure 6:**
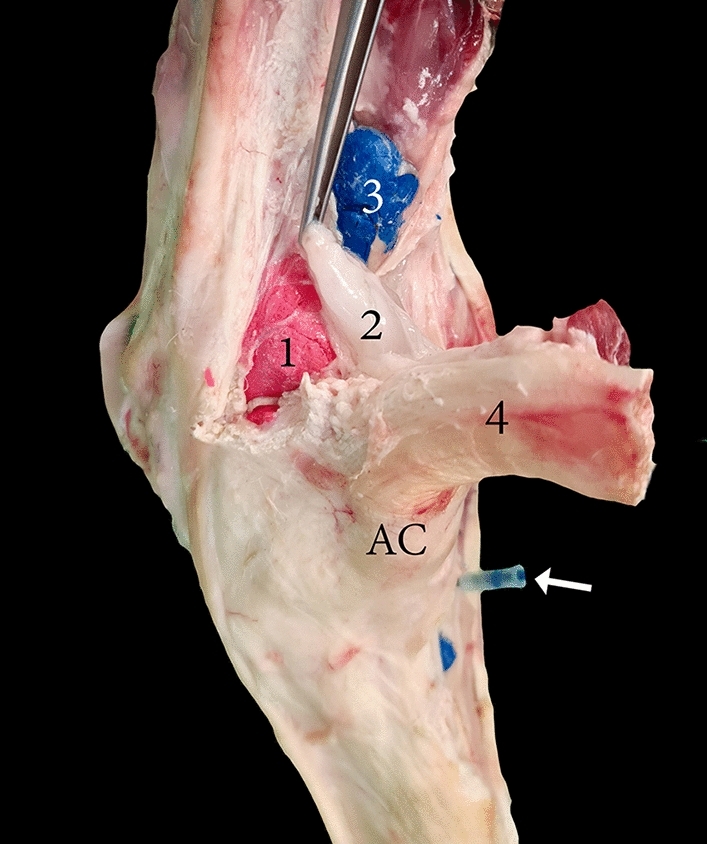
Palmar view of the left carpal joint showing 1, the lateropalmar pouch of the radiocarpal joint; 2, the subtendinous bursa; 3, inadvertent communication created with the tendon sheath of the deep digit flexor muscle (DDF); 4, extensor carpi ulnaris; AC, accessory carpal bone. The white arrow indicates the palmar injection approach to the carpometacarpal joint, which caused an inadvertent communication between the joint cavity and tendon sheath of the DDF.

There were no pouches found on the medial or lateral aspects of the carpus. However, a subtendinous synovial bursa was found proximal to the accessory carpal bone, located between the lateropalmar pouch and the flexor carpi ulnaris tendon. Notably, there was no communication between the subtendinous synovial bursa and the radiocarpal joint (Fig. 6).

**Figure 7:**
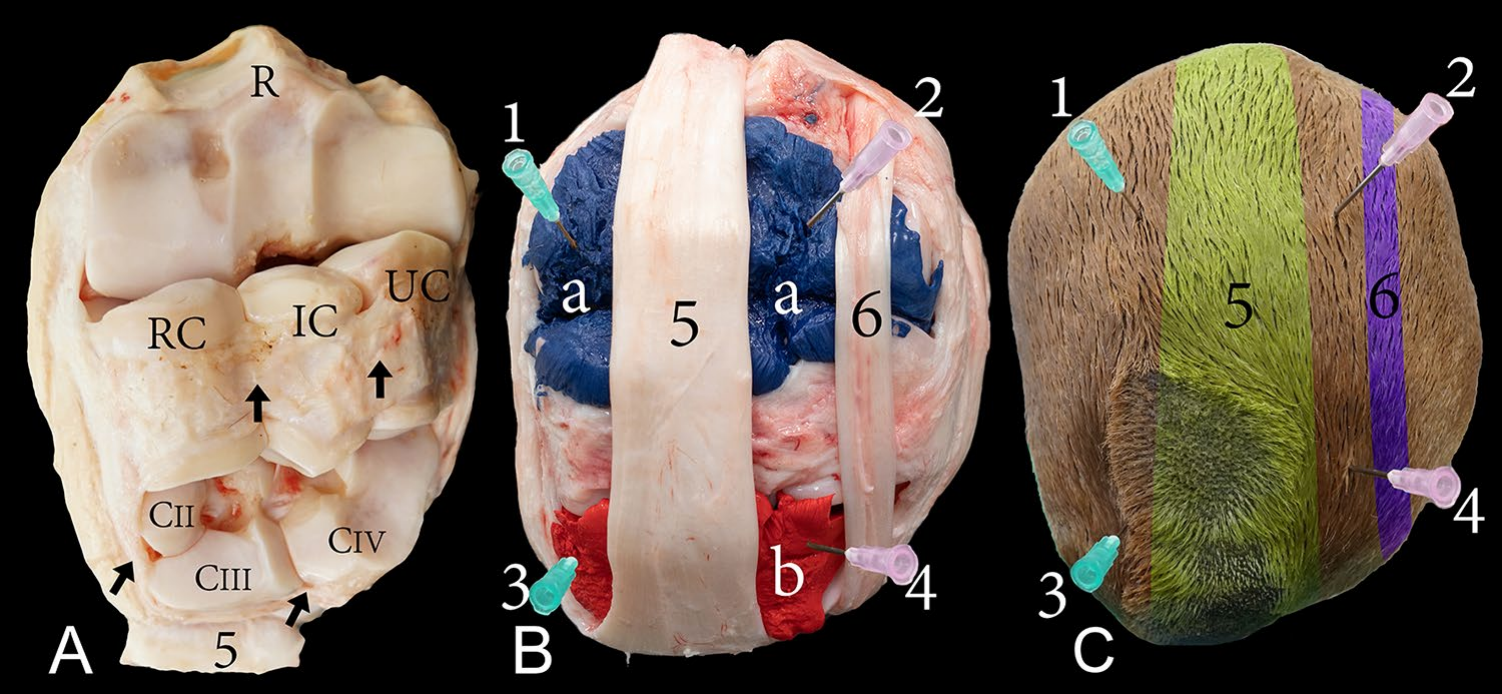
Dorsal view of a left carpal joint providing orientation for the arthrocentesis approaches through three different types of specimens: (**A**) bones, (**B**) dissected, and (**C**) intact. R, the distal end of the radius; RC, radiocarpal bone; IC, intermediate carpal bone; UC, ulna carpal bone; CII, carpal bone II; CIII, carpal bone III; CIV, carpal bone IV; a, radiocarpal joint cavity; b, intercarpal joint cavity; 1, the dorsal medial approach of radiocarpal joint; 2, the dorsal lateral approach of the radiocarpal joint; 3, the dorsal medial approach of the intercarpal joint; 4, the dorsal lateral approach of the intercarpal joint; 5, Extensor carpi radialis tendon; 6, common digital extensor tendon.

The proximal row and distal row of carpal bones are connected horizontally via short dorsal intercarpal ligaments. The distal row of carpal bones is also connected to the metacarpal bones via the dorsal carpometacarpal ligament. There are no perpendicular ligaments dorsally connecting the radius and ulna to the proximal row of carpal bones or connecting the proximal row to the distal row of carpal bones (Fig. 7A).

The tendons at the dorsal and dorsolateral aspects of the carpus are the extensor carpi radialis, abductor digiti I (*pollicis*) longus muscle, common digital extensor tendon, and the lateral digital extensor tendon (Figs. 1C, 7B). These tendons are held in place by the extensor retinaculum. The extensor carpi radialis tendon runs dorsally over the carpal joint as a broad flat tendon and inserts to the proximal dorsal surface of the metacarpal bone. It is attached to the carpal joint capsule at the distal row of carpal bones. The abductor digiti I (*pollicis*) longus muscle is enclosed by a tendon sheath, it runs across the carpal joint and the extensor carpi radialis tendon and inserts to the medial collateral ligament. The lateral and medial tendons of the common digital extensor muscle is surrounded by a common tendon sheath as it run across the carpal joint dorsolateral (Fig. 1C). The tendon of the lateral digital extensor muscle is enclosed by a separate tendon sheath and runs across the carpal joint laterally (Fig. 1C).

The tendons at the palmar aspect of the carpal joint are the extensor carpi ulnaris, flexor carpi radialis, flexor carpi ulnaris, and the deep digital flexor muscle (DDF). The tendons are held in place by the palmar retinaculum. The medial artery and median nerve are also found at the palmar aspect of the carpal joint, enclosed by the carpal canal (Fig. 3C). The extensor carpi ulnaris tendon inserts to the caudolateral surface of the metacarpal bone and accessory carpal bone and fuses with the lateral collateral ligament. After the accessory carpal bone insertion, the tendinous band continues and fuses with the interosseous medius muscle at its point of origin. The flexor carpi ulnaris tendon splits into two tendons at the level of the carpal joint. The first tendon inserts on the mediopalmar aspect of the accessory carpal bone. The second tendon fuses with the medial collateral ligament and inserts on the mediopalmar aspect of the metacarpal bone (Fig. 3C). The flexor carpi radialis tendon is enclosed in a tendon sheath as it runs through the carpal canal and inserts to the proximal palmar surface of the metacarpal bone. The DDF tendon is a combined tendon that arises from three muscle heads, it is enclosed by a synovial tendon sheath and moves through the carpal canal.

In the camel, the superficial digital flexor is tendinous. It originates from the accessory carpal bone and the metacarpal bone and runs as a flat tendon along the palmar aspect of the DDF tendon. The interosseus medius muscle is also a broad flat tendon with a palmar lateral and palmar medial origin. The lateral portion of the interosseous medius muscle originates from the ulnar carpal bone, the palmar surface of the fourth carpal bone, and the proximal palmar surface of the metacarpal bone. The medial portion originates from the radiocarpal bone and the proximal palmar surface of the metacarpal bone. It runs directly palmar to the metacarpal bone and splits distally to insert to the proximal sesamoid bones.

### Arthrocentesis

The dorsomedial and dorsolateral approach was easily accessible when the carpal joint was flexed (Fig. 7). The dorsomedial and dorsolateral arthrocentesis procedure applied directly next to the extensor carpi radialis tendon created an inadvertent communication between the radiocarpal or intercarpal joint cavity and the extensor carpi radialis tendon sheath. However, insertion of the needle 5 mm medial to the extensor carpi radialis tendon or 5–6 mm lateral to the extensor carpi radialis tendon did not lead to the creation of inadvertent communication. Other soft tissue structures were not affected by these approaches. However, the cartilage of the distal articulating surface of the radius was exposed to the needle when injecting the radiocarpal joint. Although to a lesser extent, the cartilage of the distal articulation surface of the proximal row of carpal bones and that on the proximal surface of the distal row of carpal bones were noticeably exposed to the needle when injecting the intercarpal joint cavity (Fig. 7A).

**Figure 8:**
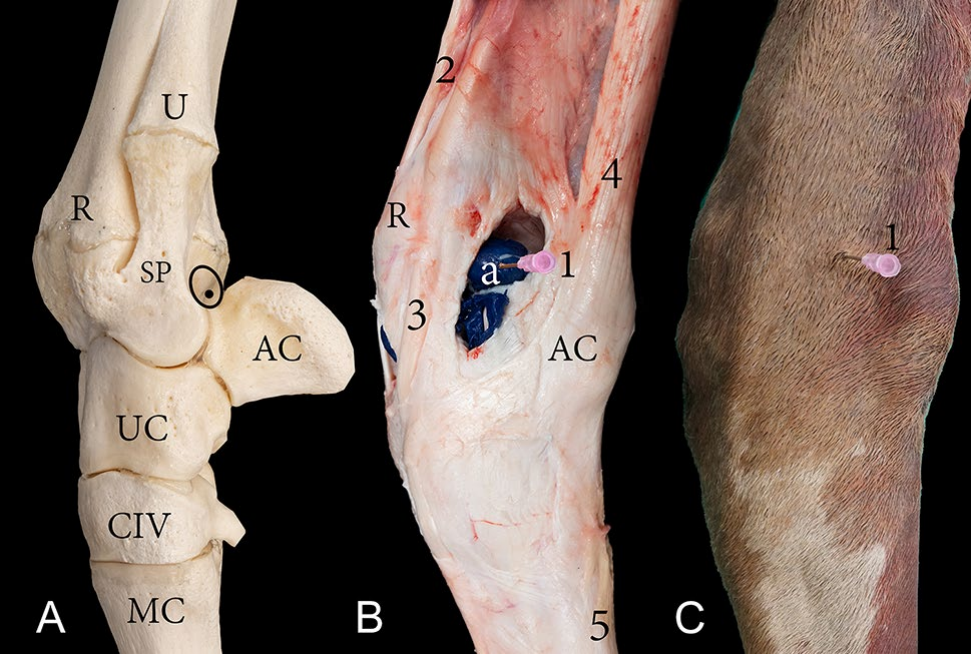
Lateral view of a left carpal joint providing orientation for the arthrocentesis approaches through three different types of specimens: (**A**) bones, (**B**) dissected, and (**C**) intact. R, the distal end of the radius; U, the distal end of the ulna; SP, lateral styloid process; AC, accessory carpal bone; UC, ulna carpal bone; CIV, carpal bone IV; MC, metacarpal bone; a, lateropalmar pouch of the radiocarpal joint; 1, the lateral approach of the radiocarpal joint; 2, extensor carpi radialis tendon; 3, lateral digital extensor tendon; 4, extensor carpi ulnaris tendon. The circle and black dot in (**A**) indicate the injection site to the lateropalmar pouch located in the impression formed between the lateral styloid process and the accessory carpal bone.

**Figure 9:**
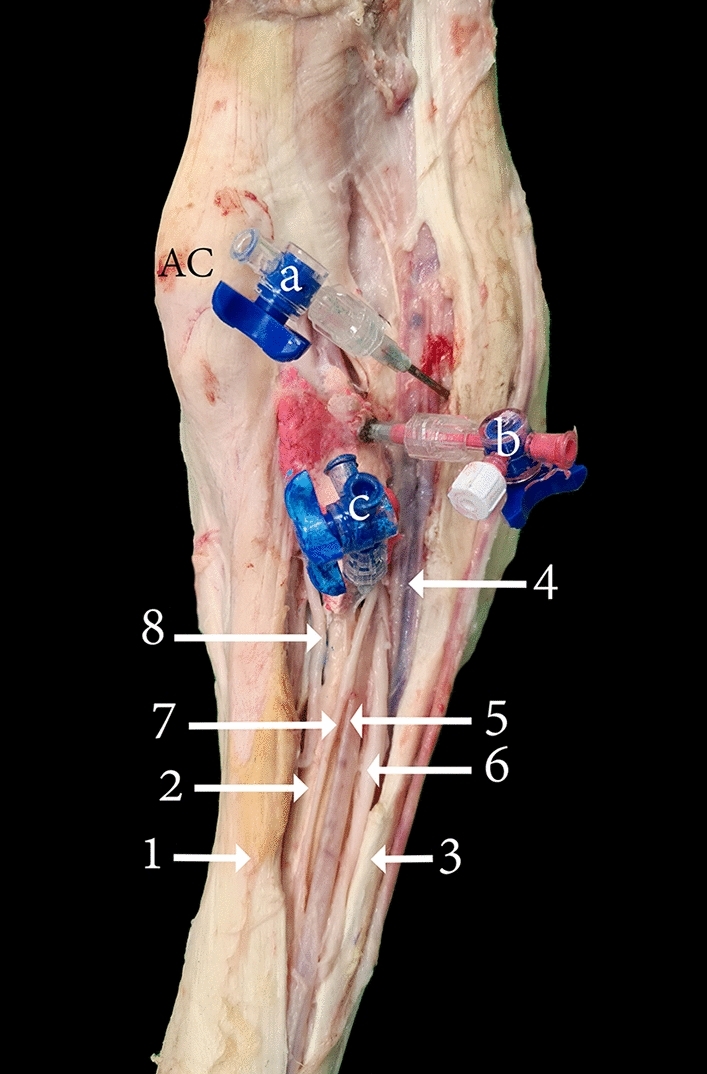
Palmar view of the left carpal joint, showing (a) the palmar medial and (b) palmar lateral arthrocentesis approach for the radiocarpal joint, and (c) the palmar approach for the carpometacarpal joint. AC, accessory carpal bone; 1, superficial digital flexor muscle; 2, deep digital flexor tendon; 3, interosseous medius muscle; 4, median vein; 5, median artery; 6, common palmar nerve of digit III; 7, common palmar nerve of digit IV; 8, ulnar nerve.

Arthrocentesis of the lateropalmar pouch of the radiocarpal joint was easily accessible under high-frequency ultrasound guidance through a lateral approach (Fig. 8). During this approach, tendons or cartilage were not exposed to needle injury. It was challenging to locate and inject the lateropalmar pouch when a palpation guide approach alone was used without the assistance of an ultrasound. Arthrocentesis of the radiocarpal and carpometacarpal joint through a palmar approach successfully filled the joint cavities with the casting material (Fig. 9). However, the palmar approach poses a grave risk of needle injury to the median artery, median vein, ulna nerve, DDF, common palmar nerve of digit III, and the common palmar nerve of digit IV. Furthermore, this approach also poses the risk of creating inadvertent communication with the DDF tendon sheath (Fig. 9).

### Ultrasonography

**Figure 10:**
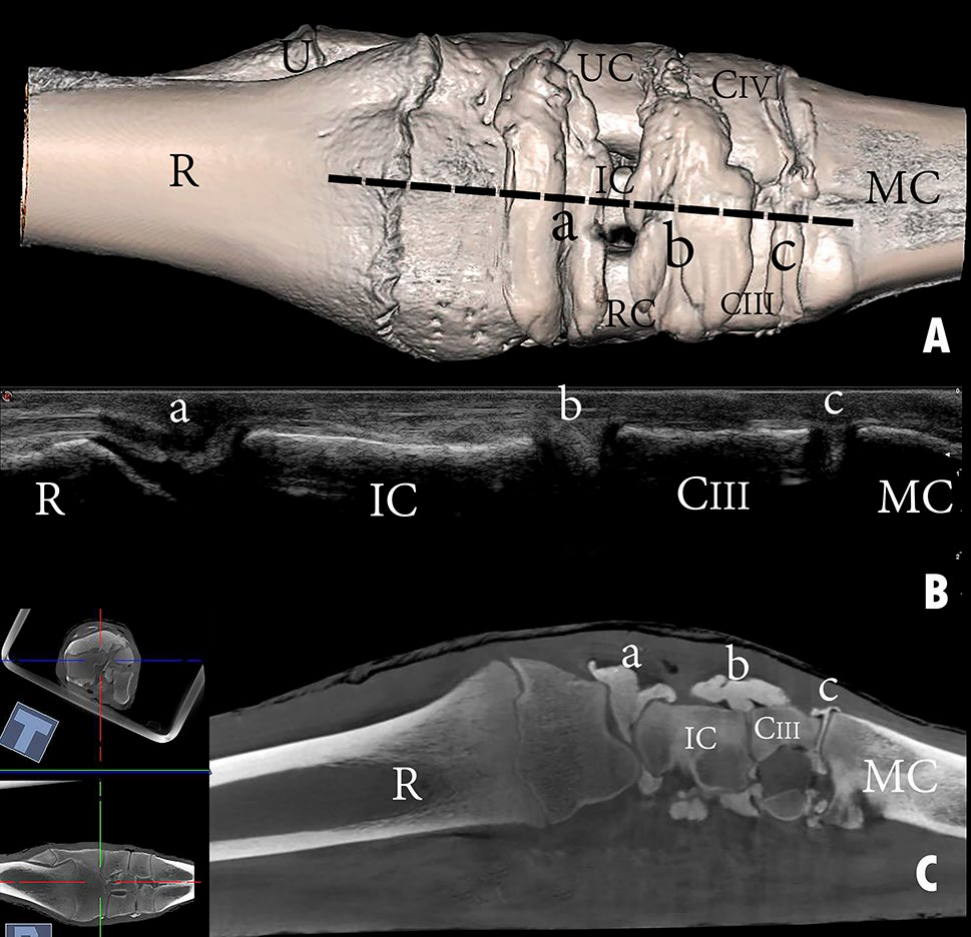
Dorsal view of a left carpal joint showing (**A**) three-dimensional reconstruction of the carpal joint (the black dotted line indicates the dorsal placement of the ultrasound probe); (**B**) longitudinal composed ultrasound image of the carpal joint; (**C**) sagittal plane computerized tomography image. R, the distal end of the radius; U, the distal end of the ulna; RC, radiocarpal bone; IC, intermediate carpal bone; UC, ulna carpal bone; CIII, carpal bone III; CIV, carpal bone IV; MC, metacarpal bone; a, radiocarpal joint cavity; b, intercarpal joint cavity; c, carpometacarpal joint cavity.

**Figure 11:**
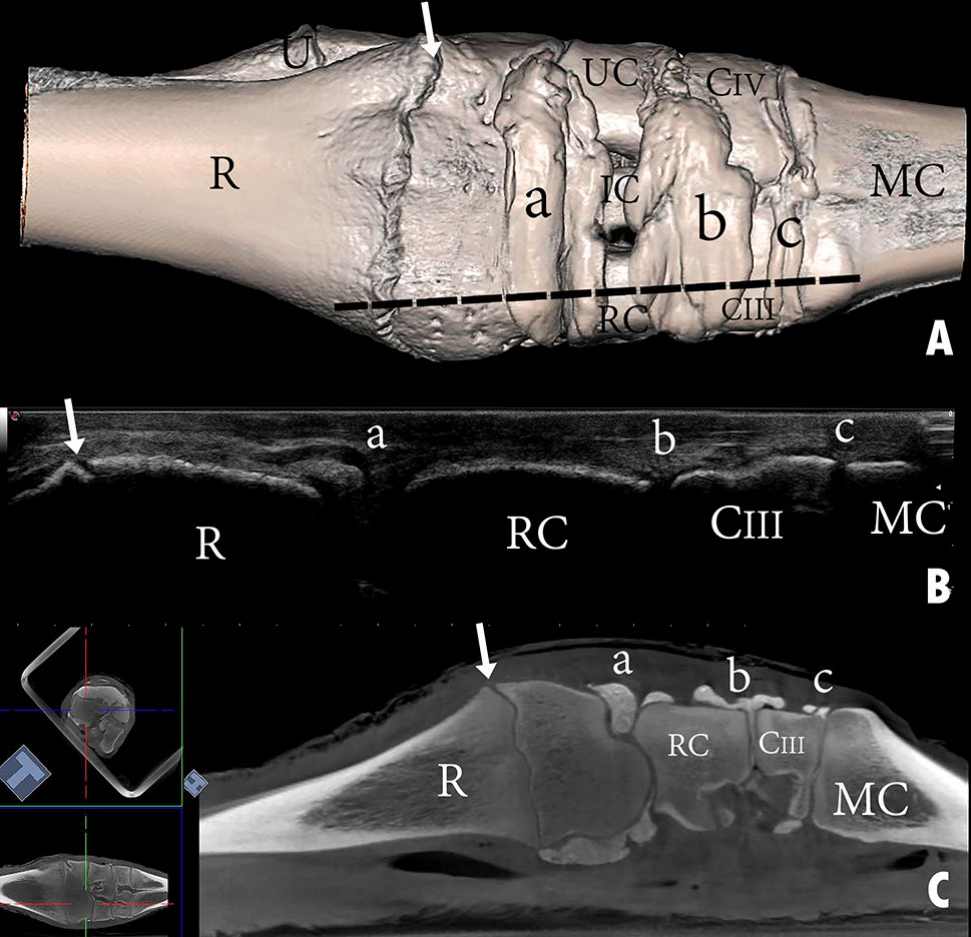
Dorsal view of a left carpal joint showing (**A**) three-dimensional reconstruction of the carpal joint (the black dotted line indicates the dorsomedial placement of the ultrasound probe); (**B**) longitudinal composed ultrasound image of the carpal joint; (**C**) sagittal plane computerized tomography image. R, the distal end of the radius; U, the distal end of the ulna; RC, radiocarpal bone; IC, intermediate carpal bone; UC, ulna carpal bone; CIII, carpal bone III; CIV, carpal bone IV; MC, metacarpal bone; a, radiocarpal joint cavity; b, intercarpal joint cavity; c, carpometacarpal joint cavity. The white arrow indicates the epiphysial line.

**Figure 12:**
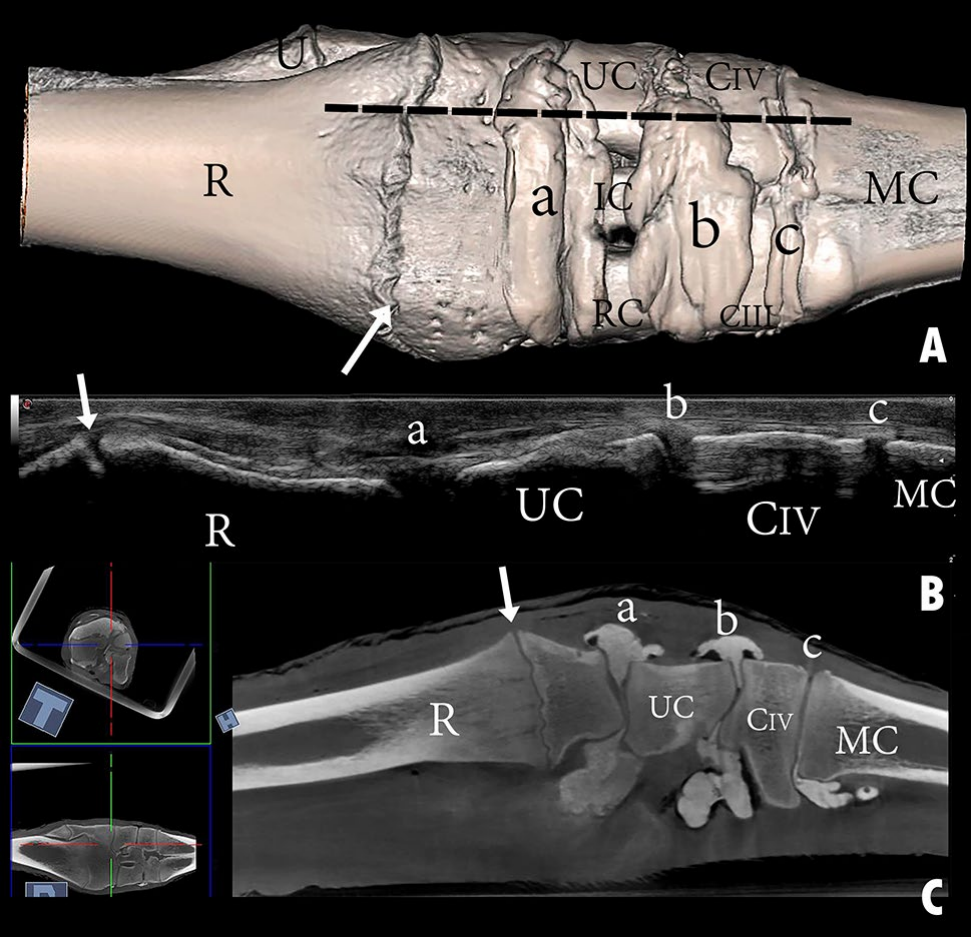
Dorsal view of a left carpal joint showing (**A**) three-dimensional reconstruction of the carpal joint (the black dotted line indicates the dorsolateral placement of the ultrasound probe); (**B**) longitudinal composed ultrasound image of the carpal joint; (**C**) sagittal plane computerized tomography image. R, the distal end of the radius; U, the distal end of the ulna; RC, radiocarpal bone; IC, intermediate carpal bone; UC, ulna carpal bone; CIII, carpal bone III; CIV, carpal bone IV; MC, metacarpal bone; a, radiocarpal joint cavity; b, intercarpal joint cavity; c, carpometacarpal joint cavity. The white arrow indicates the epiphysial line.

In the composed ultrasonography images, the soft tissue and bone surfaces displayed hyperechoic, and the bones and synovial fluid inside the joint cavities displayed hypoechoic. The carpal joint was ultrasonographically examined in dorsal (Fig. 10), dorsomedial (Fig. 11), and dorsolateral (Fig. 12) aspects in an extended and partially flexed position. In both the extended and partially flexed positions, the epiphysial line, distal end of the radius and ulna, radiocarpal joint cavity, proximal row of carpal bones, intercarpal joint cavity, distal row of carpal bones, and metacarpal bones can be clearly distinguished. In the flexed position, the radiocarpal and intercarpal joint space was increased. However, the joint space of the carpometacarpal does not increase notably during partial flexion of the carpus.

**Figure 13:**
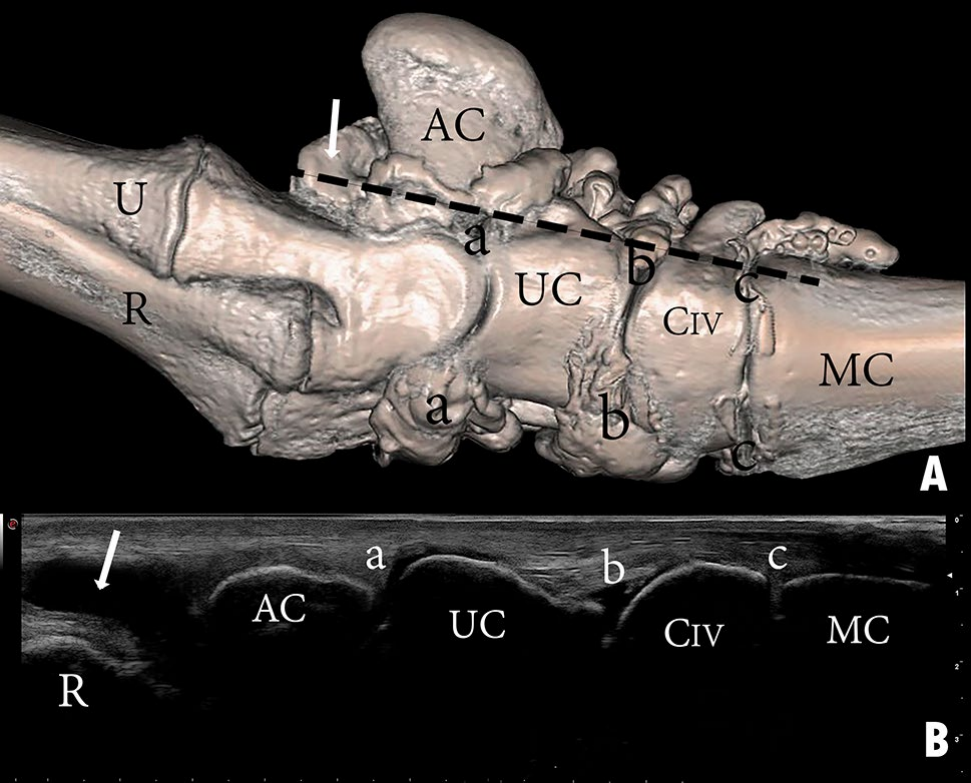
Lateral view of a left carpal joint showing (**A**) three-dimensional reconstruction of the carpal joint (the black dotted line indicates the lateral placement of the ultrasound probe); (**B**) longitudinal composed ultrasound image of the carpal joint. R, the distal end of the radius; U, the distal end of the ulna; AC, accessory carpal bone; UC, ulna carpal bone; CIV, carpal bone IV; MC, metacarpal bone; a, radiocarpal joint cavity; b, intercarpal joint cavity; c, carpometacarpal joint cavity. The white arrow indicates the lateropalmar pouch of the radiocarpal joint.

The lateral aspect of the carpus was examined using an extended joint. At the distal end of the ulna, proximal to the accessory carpal bone, the lateropalmar pouch of the radiocarpal joint was identified as it extended proximally. The radiocarpal joint cavity, ulna carpal bone, intercarpal joint cavity, carpal bone IV, the carpometacarpal joint cavity, and the metacarpal bone were also clearly displayed (Fig. 13).

**Figure 14:**
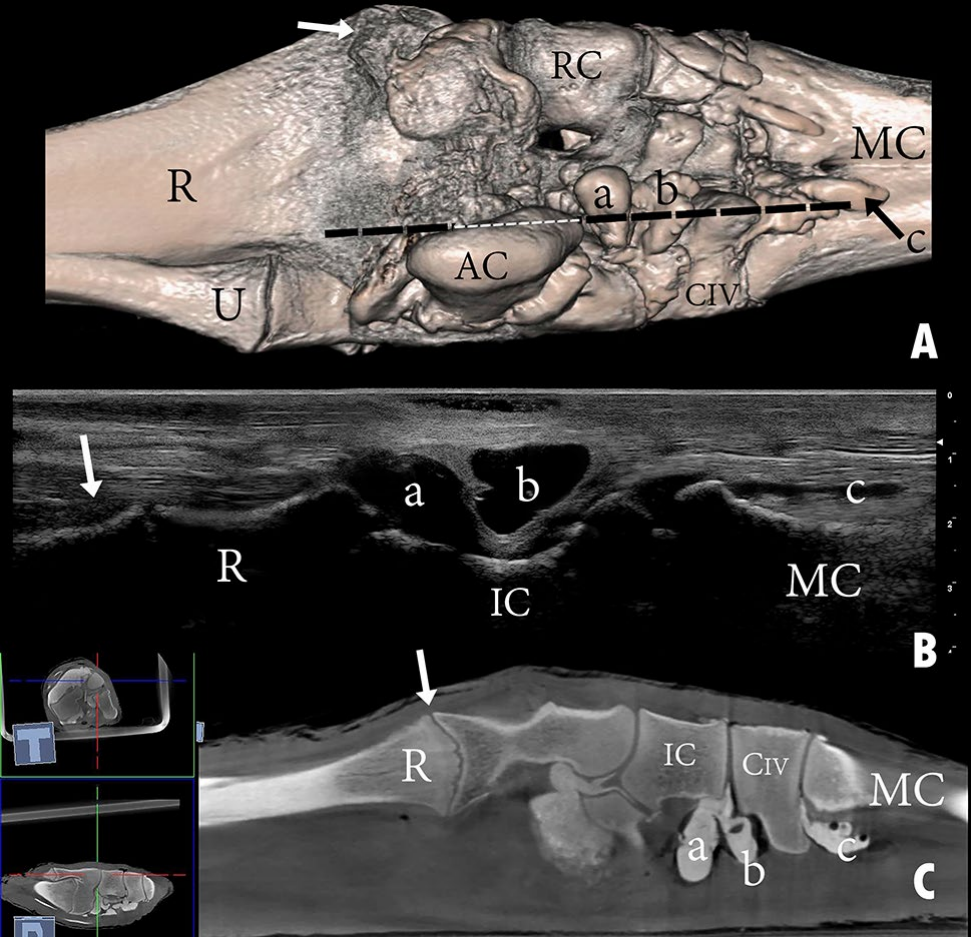
Palmar view of a left carpal joint showing (**A**) three-dimensional reconstruction of the carpal joint (the black dotted line indicates the palmolateral placement of the ultrasound probe); (**B**) longitudinal composed ultrasound image of the carpal joint; (**C**) sagittal plane computerized tomography image. R, the distal end of the radius; U, the distal end of the ulna; AC, accessory carpal bone; RC, radiocarpal bone; IC, intermediate carpal bone; CIV, carpal bone IV; MC, metacarpal bone; a and b, palmar pouch of the intercarpal joint; c, lateral pouch of the carpometacarpal joint. The white arrow indicates the epiphysial line.

**Figure 15:**
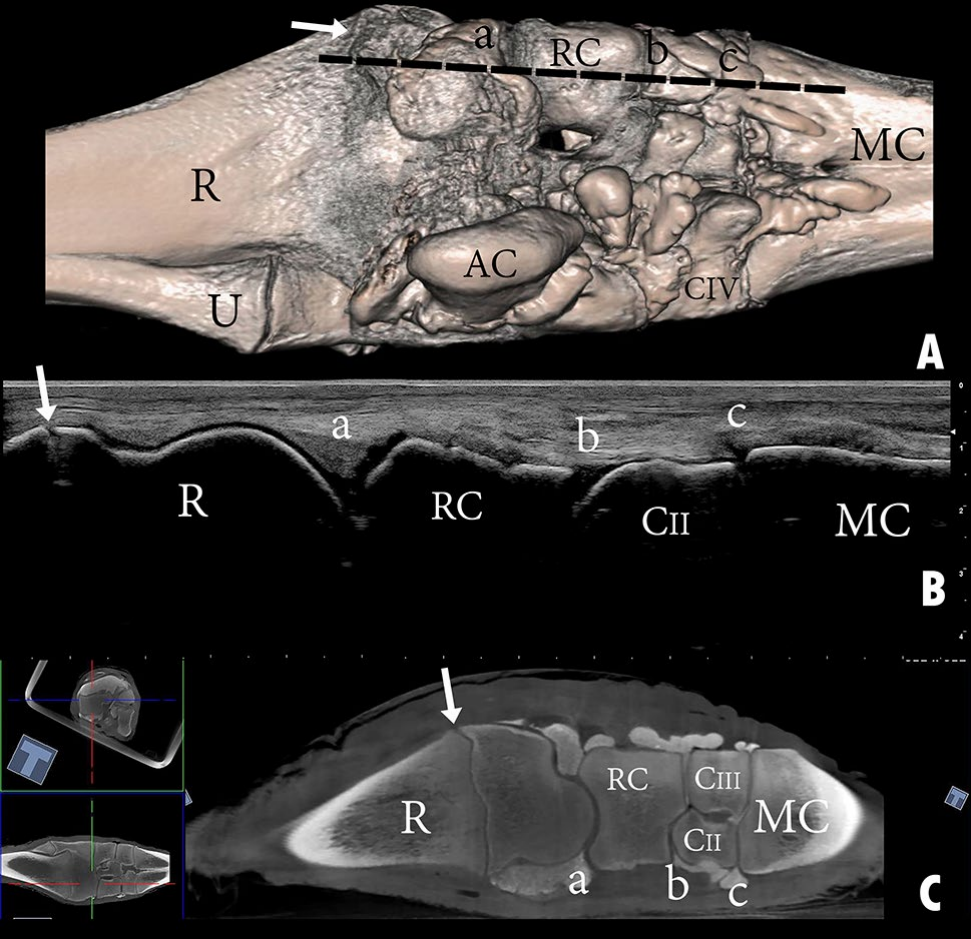
Palmar view of a left carpal joint showing (**A**) three-dimensional reconstruction of the carpal joint (the black dotted line indicates the palmomedial placement of the ultrasound probe); (**B**) longitudinal composed ultrasound image of the carpal joint; (**C**) sagittal plane computerized tomography image. R, the distal end of the radius; U, the distal end of the ulna; AC, accessory carpal bone; RC, radiocarpal bone, CII, carpal bone II; CIII, carpal bone III; CIV, carpal bone IV; MC, metacarpal bone; a, radiocarpal joint cavity; b, intercarpal joint cavity; c, carpometacarpal joint cavity. The white arrow indicates the epiphysial line.

The palmolateral (Fig. 14) and palmomedial (Fig. 15) aspects were examined using a fully extended joint. The palmolateral aspect depicts a clear view of the radiocarpal joint’s lateropalmar pouch and the intercarpal joint’s palmar pouch (Fig. 14). The distal end of the radius and ulna, ulnar carpal bone, carpal bone IV, carpometacarpal joint cavity, and metacarpal bones are viewed through this aspect. Furthermore, the pouch, which originates from the intercarpal joint and extends distally between the two origins of the lateral part of the interosseous medius muscle, was also viewed through this aspect (Fig. 14).

### Computed tomography

The reconstructed three-dimensional images of the carpal joint provided a clear view of the dorsal joint recesses, the mediopalmar and lateropalmar pouches of the radiocarpal joint, the palmar pouch of the intercarpal joint, and the medial and lateral pouches extending from the carpometacarpal joint. The CT images supported the findings yielded by the casted and macerated samples, and the CT results were consistent with those obtained through ultrasonography.

## Discussion

It was found in this study that the joint capsules of the carpal joint are systematically structured in dromedaries. The joint capsules have numerous well-structured insertion points along the carpal joint. The fibrous layer of the joint capsule distinctly inserts on the radius and ulna, the proximal and distal row of carpal bones, and the metacarpal bone, thereby supporting the stability of the joint capsule. The dissection results demonstrated that the joint capsule is further reinforced dorsally and palmarly by the extensor and flexor retinaculum. These results contradict Kassab^6^, who reported that a common joint capsule encloses the dromedary carpus, similar to what is found in the equine carpal joint^14,16^.

The internal synovial membrane of the joint capsule forms distinct folds, which are exceptionally well developed at the level between the articulating bones. These folds were found moulded into the casting material (Fig. 2). It is hypothesized that these folds allow expansion of the joint capsule during extensive flexion of the carpal joint, which occurs while the camel is sitting down. The findings of this study revealed that these folds stretch out and decrease in size in the flexed position compared with the extended position. Another function of these folds is to increase the internal surface of the synovial capsule, which would be directly related to an increase in the total number of synovial-producing cells; in turn, this process leads to an increase in the production of synovial fluid. The joint capsule inserts in such a manner that it allows synovial recesses to be formed on the radius and ulna, the proximal and distal row of carpal bones in an extended position. These recesses will allow easy displacement of synovial fluid during extension and excessive flexion of the carpal joint.

The casted samples, ultrasonography images, and CT results revealed numerous dorsal recesses and palmar pouches formed by the carpal joint capsule. Badawy et al.^4^ reported the formation of a singular dorsal recess under the extensor retinaculum, and Alsafy et al.^14^ referred to a singular palmar pouch extending from the radiocarpal joint. These researchers did not provide further details regarding the mentioned dorsal recesses or palmar pouch. However, the results of this study revealed the formation of two large pouches, namely the mediopalmar and lateropalmar pouches of the radiocarpal joint, instead of a single large palmar pouch. Furthermore, this study also provides evidence of a large palmar pouch formed by the intercarpal joint and two distinctive pouches originating from the carpometacarpal joint, extending distally between the two origins of the interosseous muscle on each side of the palmar surface of the metacarpal bone. The current conjecture is that the function of these two pouches is to facilitate circulation of synovial fluid as pressure is applied to the pouches by the contraction and relaxation of the interosseous medius muscle during movement of the forelimb.

Furthermore, it is critical to consider that any injury at this level will automatically implicate and expose the carpometacarpal and intercarpal joint cavities due to the direct connections. In the case of an open wound at this level, it should be considered that the presence of a bacterial infection will automatically affect the carpometacarpal and intercarpal joint cavities. Hence, treatment should target the whole joint rather than being limited to this area alone.

The present findings are consistent with those reported by Raza et al.^17^ and Badawy et al.^4^. According to the data, the dorsomedial and dorsolateral injection approaches are straightforward for the radiocarpal and intercarpal joints. Moreover, they are similar to the dorsal approach used in bovines, as reported by Nuss et al.^18^. The dorsomedial and dorsolateral approaches can also be successfully used for arthroscopy. However, during arthrocentesis, inadvertent communication with the extensor carpi radialis tendon sheath can be created via the dorsomedial and dorsolateral approaches. This study also revealed that the dorsolateral approach could create inadvertent communication between the joint cavities and the common digital extensor tendon sheath. Alsobayil et al.^19^ reported an existing communication between the radiocarpal joint and the tendon sheath of the extensor carpi radialis, and laterally, a communication with the common digital extensor muscle. However, the investigators of this study concluded that a communication only exists when the needle is inserted through the tendon sheath of these two muscles during the arthrocentesis procedure. These findings were further supported by the results obtained from the samples injected using a palmar approach. The palmar approach samples did not show communication between the joint cavities and the tendon sheath of the extensor carpi radialis tendon or the common digital extensor tendon. The absence of an exciting communication between the extensor carpi radialis tendon sheath and the radiocarpal joint is a similarity shared by bovines and dromedaries^18^. Badawy et al.^4^ recommended that the point of needle insertion should be 2–3 mm from the extensor carpi radialis tendon during the dorsomedial and dorsolateral approaches. Nonetheless, the results of this study indicated that the point of insertion should be approximately 5 mm medial and 5–6 mm lateral to the extensor carpi radialis tendon during the dorsomedial and dorsolateral approach, respectively, to avoid any inadvertent communication. Alternatively, during the dorsolateral approach, the extensor carpi radialis tendon and common digital extensor tendon can be palpated, and the needle can be inserted midway between these two tendons to prevent inadvertent communication with either of the tendon sheaths.

The risk of injury to the articulating cartilage surface of the radiocarpal and intercarpal joint during arthrocentesis via a dorsal approach remains of grave concern. The absence of perpendicular ligaments between the radius, proximal row of carpal bones, and the distal row of carpal bones allows extensive flexion in the radiocarpal and intercarpal joint region. The extent to which the radiocarpal and intercarpal joint flexes leads to excessive cartilage exposure, rendering the dorsomedial and dorsolateral approaches hazardous (Fig. 7A). On the other hand, the carpometacarpal joint cannot be injected directly via a dorsal approach. The short dorsal ligaments between the distal row of carpal bones and the metacarpal bone limit the range of flexion of the carpometacarpal joint, thereby preventing the insertion of a needle dorsally into the joint cavity.

Through a lateral approach, arthrocentesis of the lateropalmar pouch of the radiocarpal joint is a feasible alternative to the dorsomedial and dorsolateral approaches. The risk of forming inadvertent communication with the tendon sheaths, as well as that of injury to the articulating cartilage surface, are eliminated. Experienced clinicians can locate the site of needle insertion through a palpation guided approach. The point of needle insertion can be located by palpating the distal end of the ulna (lateral styloid process) and locating the impression formed between the ulna and the accessory carpal bone, as illustrated in Fig. 8A. The needle should be inserted at a 90-degree angle to the sagittal plane of the carpal joint. However, in the absence of clinical arthrocentesis experience, the accuracy of the needle placement during the lateral approach is limited when only a palpation guided approach is used^4^. Ultrasound guidance is a feasible solution to improve the accuracy of needle placement.

Under ultrasound guidance, it is critical to ensure that the subtendinous synovial bursa, located between the lateropalmar pouch and the flexor carpi ulnaris tendon, is not mistaken for the joint cavity. To the best of our knowledge, this subtendinous synovial bursa has not been previously described (Fig. 6). Furthermore, in this study, no medial or lateral approach was found to be a feasible alternative injection approach for the intercarpal or carpometacarpal joint, which agrees with Alsobayil et al.^19^, and Badawy et al.^4^.

After an extensive investigation of the palmar pouches formed by the radiocarpal and intercarpal joint, no feasible palmar injection approach was identified. Arthrocentesis of the radiocarpal and carpometacarpal joint via the palmar approach successfully filled the respective joint cavities with the casting material. However, an inadvertent communication was created with the DDF tendon sheath proximally and distally (Fig. 9). This approach exposed the median vein, median artery, ulnar nerve, common palmar nerve of digit III, and the common palmar nerve of digit IV to needle injury (Fig. 9).

It has been previously reported that CT and ultrasound imaging of the dromedary carpus relates well to the gross anatomy^4,6^. The findings of this study provided evidence that CT and ultrasound imaging provides an accurate representation of the joint cavities of the carpal joint. Kassab^6^ reported difficulty during the ultrasonographic examination of amputated limbs. However, this study managed any potential difficulty of this nature by conducting the ultrasonographic examination within two hours post mortem.

## Conclusion

This study provides valuable insight regarding the anatomy and topography of the carpal joint of the dromedary supported by ultrasonography and computed tomography images. Arthrocentesis of the lateropalmar pouch of the radiocarpal joint via a lateral approach in an extended position is clinically the safest arthrocentesis approach due to a lower risk of damaging the articulation cartilage surface and the elimination of creating inadvertent communication with the extensor carpi radialis, common digital extensor, and DDF tendon sheaths. The use of a high-frequency ultrasound-guided approach is recommended to ensure accurate needle placement. When the carpal joint is flexed in the sitting position, it is recommended to use the described dorsomedial and dorsolateral approaches to the radiocarpal and intercarpal joints for arthrocentesis and arthroscopy. Importantly, the palmar injection approach for the carpal joint cavities must be avoided to prevent inadvertent communication with the tendon sheaths and needle injury to the nerves, arteries, veins, and tendons present in this area.

## Data Availability

The datasets generated during the current study are available from the corresponding author on reasonable request.
